# Outpatient intracameral viscosurgical device reinflation for early hypotony after PreserFlo MicroShunt implantation: a case series

**DOI:** 10.1016/j.ajoc.2026.102598

**Published:** 2026-05-09

**Authors:** Miho Takenaka, Yasuko Ikegami, Miwako Yoshimoto, Rei Sakata

**Affiliations:** a Department of Ophthalmology, Kameda General Hospital, Chiba, Japan; b Department of Ophthalmology, Tokyo Metropolitan Geriatric Hospital and Institute of Gerontology, Tokyo, Japan; c Department of Ophthalmology, Graduate School of Medicine and Faculty of Medicine, The University of Tokyo, Tokyo, Japan

**Keywords:** PreserFlo MicroShunt, Hypotony, Ophthalmic viscosurgical device, Anterior chamber reformation, Choroidal detachment

## Abstract

**Purpose:**

To describe a simple and reproducible stepwise outpatient management approach using intracameral high-viscosity ophthalmic viscosurgical device (OVD) reinflation for early Seidel-negative hypotony with shallow anterior chamber (AC) and choroidal detachment (CD) after PreserFlo MicroShunt implantation.

**Observations:**

This retrospective case series included three consecutive pseudophakic glaucoma eyes that developed Seidel-negative hypotony (lowest intraocular pressure [IOP] 3–5 mmHg) with shallow AC and CD in the early postoperative period after PMS implantation (including one case after bleb revision). In all eyes, a fixed initial small volume (0.2 mL) of high-viscosity or viscoadaptive sodium hyaluronate was injected intracamerally at the slit lamp or under the operating microscope to restore AC depth and IOP. AC reformation and CD resolution were achieved within 1–4 days without additional surgical intervention. No clinically evident corneal endothelial decompensation, visually significant IOP spikes (≥20 mmHg), or loss of visual acuity occurred during follow-up.

**Conclusions and Importance:**

Small-volume intracameral OVD reinflation appears to provide a simple, reproducible, and minimally invasive first-line option for early Seidel-negative hypotony after PMS implantation. A stepwise outpatient management approach may help standardize clinical decision-making for PMS-related shallow AC and CD, potentially reducing the need for reoperation while maintaining IOP control.

## 1 Introduction

The PreserFlo MicroShunt (PMS) has gained popularity as a minimally invasive approach for glaucoma surgery. In the postoperative period, hypotony due to overfiltration may occur (1.7 %–39 %), occasionally accompanied by a shallow anterior chamber (AC) and choroidal detachment (CD). When wound leakage is excluded, this complication is thought to result from a mismatch between device outflow and the resistance of surrounding tissues. Therefore, management is focused on two main strategies: restoring AC volume and intraocular pressure (IOP) and modulating excessive filtration.

In incisional glaucoma surgery, AC reformation using an ophthalmic viscosurgical device (OVD), such as Healon, has long been recognized as effective, and its clinical utility has been further supported in recent years.^1^, ^2^, ^3^, ^4^^)^ Additional evidence includes slit-lamp reformation in the outpatient setting, widespread adoption among American Glaucoma Society members, and findings from randomized controlled trials.^5^^,^^6^^)^ Variations in OVD concentration and administration route have been explored, broadening its application from early to chronic hypotony, including severe cases.^7^, ^8^, ^9^^)^ Although the underlying mechanism of Seidel-negative hypotony with a shallow AC and/or CD (i.e., an imbalance between aqueous outflow and tissue resistance/aqueous production) is shared across bleb-forming procedures and other devices such as tube shunts or gel stents, postoperative decision-making after PMS implantation involves practical PMS-specific considerations. These include early tissue–device interactions affecting outflow and, in some eyes, the use and subsequent removal of intraluminal stenting, which can introduce distinct timing and escalation decisions in the early postoperative period. Therefore, a structured outpatient, stepwise first-line approach tailored to the PMS postoperative setting has not been well described.

In this report, we describe three consecutive cases of early Seidel-negative hypotony with shallow AC and CD after PMS implantation that were successfully managed using a small-volume intracameral high-viscosity OVD reinflation approach, and we introduce a practical stepwise outpatient approach that may serve as a reproducible first-line strategy for this complication.

## 2 Findings

Details of the three cases are summarized in Table 1. Written informed consent was obtained from all patients for publication of this case series and the associated clinical images. Our study was performed in accordance with the Declaration of Helsinki. The institutional review board of Tokyo Metropolitan Geriatric Hospital approved the study protocol.

**Table 1:** Summary of cases and timing of interventions.

Variable	Case 1	Case 2	Case 3
Age/sex	84/F	88/M	62/M
Dx	XFG	OAG	XFG
Intraluminal stent suture placement removal (POD)	Yes (23)	No	No
MMC	0.04%/3 min	0.05%/1.5 min	0.04%/3 min; revision
Onset of hypotony (POD)^a^	29	3	6
Prior OVD (PODs)	33, 40, 47 (DisCoVisc/Viscoat/DisCoVisc)	None	None
Our OVD (POD/type/mL)	89; Healon5; 0.2	7; Healon; 0.2	13; Healon; 0.2
Minimum IOP before OVD reinflation (mmHg)	3	3	5
AC depth (Van Herick grade)	Deep	VH2	VH2
Extent of choroidal detachment (quadrants)	2	2	4
IOP 24 h (mmHg)	18	5	5
Time to CD resolution (days)	3	7	4
Last follow-up (months)	1	6	4
Spike ≥20 (mmHg)	No	No	No
Preoperative BCVA/Worst BCVA during hypotony/BCVA at last follow-up	0.15/0.52/0.4	0.3/0.52/0.4	0.15/0.3/0.0

Prior OVD indicates intracameral OVD injections at the referring clinic before transfer. In Case 1, the extent of CD may have been underestimated due to poor dilation.

Abbreviations: M, male; F, female; Dx, diagnosis; XFG, exfoliation glaucoma; OAG, open-angle glaucoma; POD, postoperative day; MMC, mitomycin C; OVD, ophthalmic viscosurgical device; IOP, intraocular pressure; AC, anterior chamber; CD, choroidal detachment; BCVA, best-corrected visual acuity.

a Onset POD indicates the day when hypotony was first documented. This series focuses on the early postoperative period (≤30 days after PreserFlo MicroShunt implantation). In Case 1, CD was confirmed on POD 29 (6 days after intraluminal stent removal on POD 23); standardized OVD reinflation at our institution was performed on POD 89 due to delayed referral.

This was a retrospective case series of three consecutive eyes that developed hypotony after PMS implantation. The PMS was inserted in the superonasal or superotemporal quadrant in all eyes, and mitomycin C (MMC) was applied using MMC-soaked sponges placed in the subconjunctival/Tenon's pocket over the intended bleb area, followed by copious irrigation with balanced salt solution. The MMC concentration/exposure time were 0.04% for 3 minutes in Cases 1 and 3 and 0.05% for 1.5 minutes in Case 2; in Case 3, additional MMC was used at bleb revision (Table 1). Postoperatively, patients received 0.1% betamethasone and moxifloxacin ophthalmic solutions four times daily. Hypotony was managed using a structured stepwise outpatient approach.

## 3 Stepwise management approach

For eyes presenting with early postoperative Seidel-negative hypotony with a shallow AC and/or CD after PMS implantation, the following stepwise management approach was applied. Initial assessment included best-corrected visual acuity, slit-lamp evaluation of AC depth, Seidel testing, fundus examination and B-scan ultrasonography to assess the extent of CD, and macular optical coherence tomography when indicated (e.g., decreased vision or suspected hypotony maculopathy). Conservative measures were initiated, including cycloplegia, rest, and a stepwise increase in topical steroids, while excluding an overt wound leak. If hypotony with a shallow AC and/or CD persisted after conservative measures, or if there was concern for vision-threatening anterior segment/posterior segment complications (e.g., progressive iridocorneal touch/corneal risk, decreased visual acuity or macular folds), small-volume intracameral OVD reinflation was performed as a first-line intervention. A fixed initial volume of 0.2 mL of high-viscosity or viscoadaptive sodium hyaluronate (Healon or Healon5) was injected intracamerally at the slit lamp or under the operating microscope, aiming to reform the AC and avoid excessive postoperative IOP elevation.

Patients were re-evaluated within 24–72 hours to document IOP, AC configuration, and CD status. If AC depth and CD resolution were maintained, routine follow-up was continued. In the event of recurrence, one repeat OVD reinflation was allowed. Persistent hypotony/shallow AC/CD despite reinflation, or repeated recurrence within 72 hours after the repeat injection, prompted escalation to filtration-modulating interventions (e.g., intraluminal stenting of the PMS when appropriate, compression sutures, or tube ligation) rather than continued outpatient reinflation alone.

Inclusion criteria were onset of hypotony (IOP ≤5 mmHg) with shallow AC and/or CD during the early postoperative period (≤30 days after PMS implantation) and a negative Seidel test. In Case 1, hypotony developed after intraluminal stent removal on POD 23, and CD was first documented on POD 29; the condition persisted despite conservative management and prompted multiple intracameral OVD attempts at the referring clinic before referral. Shallow AC was defined as a peripheral AC depth of ≤1/4 corneal thickness (Van Herick grade ≤1) and/or any iridocorneal touch. ‘Moderately shallow’ AC was defined as a peripheral depth of 1/4–1/2 corneal thickness (Van Herick grade 2) without iridocorneal touch. CD was confirmed by fundus examination. In all cases, a fixed small volume of OVD (0.2 mL) was injected into the AC at the slit lamp or under the operating microscope, according to the surgeon's preference. The step-by-step technique of intracameral OVD reinflation used in this series is shown in Supplementary Video 1.

## 4 Case 1

An 84-year-old pseudophakic woman with exfoliation glaucoma underwent PMS implantation with prophylactic intraluminal stenting at another clinic. Preoperatively, her IOP was 30 mmHg on 5 medications, and best-corrected visual acuity (BCVA) was 0.15 logMAR. A 10-0 nylon intraluminal stent placed at the time of PMS implantation was removed on POD 23. After stent removal, hypotony developed (IOP 3 mmHg) with CD involving 2 quadrants, despite a deep AC (POD 29). At the referring clinic, intracameral OVD (DisCoVisc/Viscoat/DisCoVisc) injection was attempted three times (POD 33, 40, and 47), resulting in only transient AC deepening without sustained CD resolution, and she was subsequently referred to our hospital. Conservative measures (cycloplegia and rest) were continued for 12 days without improvement.

On presentation, the Seidel test was negative and the bleb was diffuse without a focal leak. Due to delayed referral and a protracted course, standardized OVD reinflation at our institution was performed on POD 89. Given persistent hypotony with CD and decreased VA, intracameral reinflation with a viscoadaptive OVD (Healon5, 0.2 mL; Johnson & Johnson Vision) was performed under an operating microscope. The AC deepened immediately with stable configuration and no iridocorneal touch. At 24 hours, IOP was 18 mmHg with improving CD, and CD resolved within 3 days. No further surgical intervention was required. During 1 month of follow-up, no excessive IOP spike (≥20 mmHg) occurred and no evident corneal endothelial damage was observed; BCVA at last follow-up was 0.4 logMAR.

## 5 Case 2

An 88-year-old pseudophakic man with open-angle glaucoma underwent PMS implantation without intraluminal stenting. Preoperatively, IOP was 23 mmHg on three medications, and BCVA was 0.3 logMAR. On POD 3, IOP was 3 mmHg, and on POD 7 it remained 4 mmHg with a shallow AC (Van Herick grade 2) and CD involving two quadrants, confirmed on fundus examination. BCVA decreased from 0.3 to 0.52 logMAR. Macular OCT showed no hypotony maculopathy, and there was no iridocorneal touch.

Conservative measures (cycloplegia, rest, and stepped-up topical steroids) were initiated for 4 days without sufficient improvement. The Seidel test was negative and the bleb was diffuse without a focal leak. Given persistent hypotony with shallow AC and CD, intracameral injection of a high-viscosity OVD (Healon, 0.2 mL; Johnson & Johnson Vision), as shown in Supplementary Video 1, was performed under an operating microscope. The AC reformed immediately. Immediate post-injection IOP was not measured; IOP was assessed at 24 hours. At 24 hours, IOP was 5 mmHg with improving CD, and CD resolved within 7 days. No further intervention was required, and no excessive IOP spike (≥20 mmHg) occurred during 6 months of follow-up; BCVA at last follow-up was 0.4 logMAR.

## 6 Case 3

A 62-year-old pseudophakic man with exfoliation glaucoma underwent PMS implantation followed by bleb revision for scarring. Preoperatively, IOP was 24 mmHg on four medications, and BCVA was 0.15 logMAR. The bleb revision consisted of conjunctival peritomy with lysis of Tenon's adhesions with mitomycin C to restore bleb function. On POD 6, IOP decreased to 5 mmHg with a shallow AC (Van Herick grade 2) and CD involving four quadrants, confirmed on fundus examination. BCVA changed from 0.15 to 0.3 logMAR, and there was no iridocorneal touch.

**Fig. 1:**
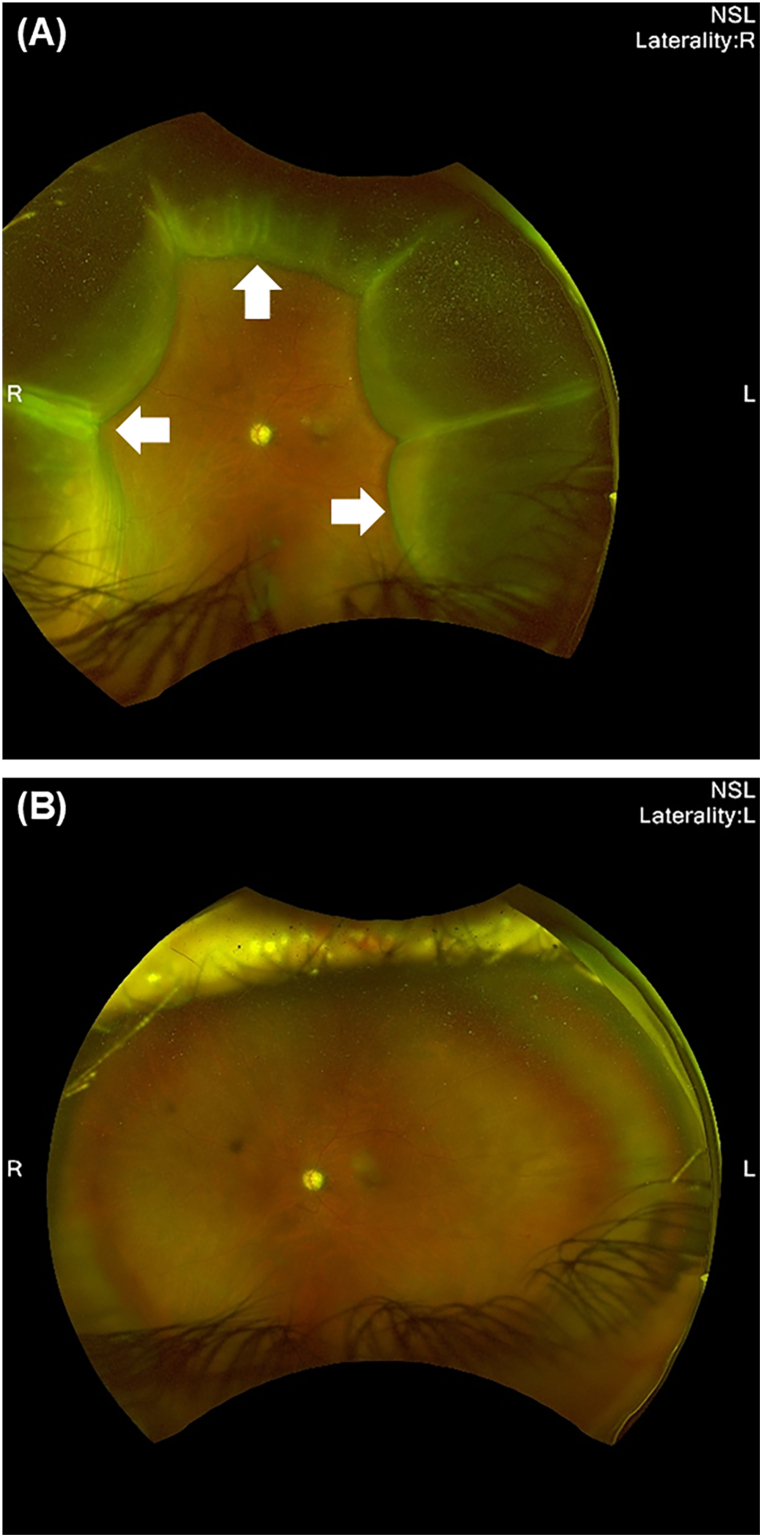
Representative case showing choroidal detachment (CD) before and after intracameral ophthalmic viscosurgical device (OVD) reinflation following PreserFlo MicroShunt implantation. (A) Color fundus photograph at presentation demonstrating choroidal detachment (arrows) in the setting of hypotony (intraocular pressure [IOP] 3 mmHg). (B) Color fundus photograph obtained three days after small-volume intracameral OVD injection, showing complete resolution of the CD and restoration of a normal choroidal contour (IOP 12 mmHg). CD, choroidal detachment; OVD, ophthalmic viscosurgical device; IOP, intraocular pressure; PMS, PreserFlo MicroShunt. (For interpretation of the references to colour in this figure legend, the reader is referred to the Web version of this article.)

Conservative measures (cycloplegia, rest, and stepped-up topical steroids) were continued for 7 days. The Seidel test was negative and the bleb was diffuse without a focal leak. Intracameral injection of a high-viscosity OVD (Healon, 0.2 mL; Johnson & Johnson Vision) was performed on POD 13 under an operating microscope, resulting in immediate AC stabilization (well-formed AC). Immediate post-injection IOP was not measured; IOP was assessed at 24 hours. At 24 hours, IOP was 5 mmHg, and CD resolved within 4 days (Fig. 1). No IOP spike (≥20 mmHg) was observed during 4 months of follow-up; BCVA at last follow-up was 0.0 logMAR.

## 7 Discussion

Across the three PMS eyes in this series, small-volume intracameral OVD reinflation consistently deepened the AC and led to complete resolution of CD within a few days, without clinically significant IOP spikes (≥20 mmHg) or the need for reoperation. Hypotony with shallow AC and CD after glaucoma filtration surgery is classically associated with advanced age, exfoliation syndrome, and intensive mitomycin C use, which alter ocular rigidity and tissue permeability; similar mechanisms likely contribute after PMS implantation, where overfiltration and temporarily reduced aqueous production result in device outflow exceeding inflow in the absence of a wound leak. Intraluminal stenting may reduce the risk of postoperative hypotony in selected eyes, but clinicians are still faced with cases that develop Seidel-negative hypotony and CD despite appropriate intraoperative measures. Published data on PMS–specific structured outpatient management of early Seidel-negative hypotony with shallow AC and CD are scarce. After conducting a literature review on 2 December 2025 utilizing PubMed, Google Scholar, and Ichushi-Web with the key words “PreserFlo MicroShunt”, “hypotony”, “shallow anterior chamber”, “choroidal detachment”, and “ophthalmic viscosurgical device”, we did not find any prior reports describing a structured outpatient stepwise approach using small-volume intracameral OVD reinflation for this scenario. Our case series, therefore provides preliminary evidence supporting this pragmatic stepwise approach.

Intracameral OVD injection addresses this condition through several mechanisms: temporary restoration of AC volume and IOP to normalize the choroidal pressure gradient; transient resistance near the outflow site to buffer filtration; and partial blockade of inflow into the device lumen. High-viscosity OVDs persist in the AC for a relatively long duration, enhancing the short-term durability of the effect.^2, 3)^ In Case 1, multiple prior intracameral OVD attempts at the referring clinic did not provide sustained resolution. In contrast, a single standardized 0.2 mL Healon5 reinflation at our institution resulted in rapid and durable AC reformation. This difference may reflect differences in OVD properties and/or timing relative to postoperative outflow stabilization after stent removal; however, this remains speculative given the retrospective nature of the case and the limited details available from the referring clinic. Clinical studies further support this approach, including outpatient slit-lamp reformation, widespread adoption in practice, and findings from randomized trials.^5)−6^).

**Fig. 2:**
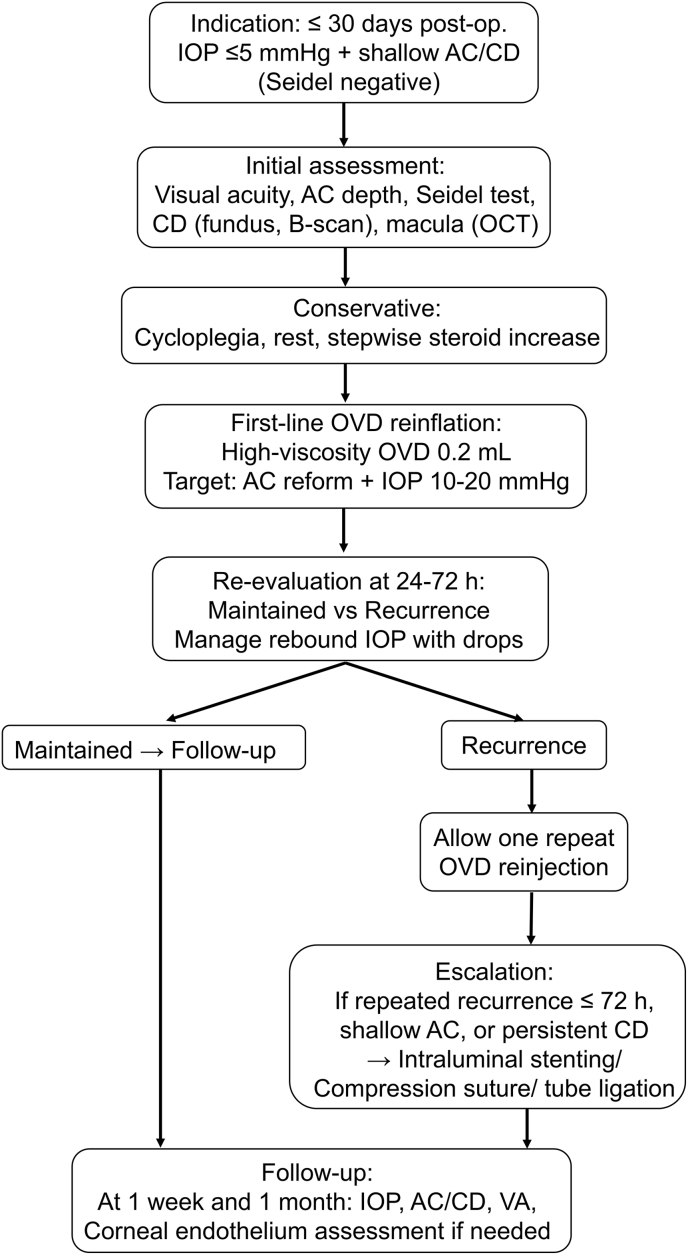
Proposed outpatient algorithm for the management of early Seidel-negative hypotony with shallow anterior chamber (AC) and choroidal detachment (CD) after PreserFlo MicroShunt (PMS) implantation. The algorithm applies to eyes in the early postoperative period (≤30 days after surgery) presenting with IOP ≤5 mmHg, shallow AC/CD, and a negative Seidel test. Initial assessment includes visual acuity, AC depth, Seidel testing, evaluation of CD by fundus examination and B-scan ultrasonography, and macular optical coherence tomography, followed by conservative measures (cycloplegia, rest, and stepwise increase in topical steroids). If hypotony and CD persist, a small-volume intracameral high-viscosity ophthalmic viscosurgical device (OVD; fixed initial volume of 0.2 mL) reinflation is performed as a first-line intervention, targeting AC reformation and an IOP of 10–20 mmHg. Patients are re-evaluated at 24–72 hours: if AC depth and CD resolution are maintained, routine follow-up is continued; in the case of recurrence, one repeat OVD reinflation is allowed. Repeated recurrence within 72 hours, persistent shallow AC, or persistent CD prompts escalation to more invasive procedures such as intraluminal stenting of the PMS, compression sutures, or tube ligation. Follow-up at 1 week and 1 month includes documentation of IOP, AC/CD status, visual acuity, and corneal endothelial status when indicated. AC, anterior chamber; CD, choroidal detachment; IOP, intraocular pressure; OVD, ophthalmic viscosurgical device; PMS, PreserFlo MicroShunt; VA, visual acuity.

However, severe IOP spikes following viscoadaptive OVD injection have been reported, emphasizing the importance of low-volume administration and close follow-up within 24–72 h.^10^^)^ Our small series aligns with previous studies on fixed-volume injection after Baerveldt implantation, demonstrating reproducible AC reformation and CD resolution with small-volume (0.2 mL) OVD injections.^11^^)^ Although OVD has been used for chronic hypotony and, in severe cases, via intravitreal administration, our proposed algorithm offers a simple, reproducible, and safe first-line strategy suitable for outpatient management (Fig. 2).^8), 9)^ Although intracameral OVD injection for hypotony has been reported after trabeculectomy and various device-based bleb-forming procedures (including gel stents and tube shunts), management approaches are heterogeneous, and the optimal outpatient timing, dosing, and escalation strategy have not been well established for the PMS postoperative setting. Importantly, we do not suggest a unique pathophysiology exclusive to PMS; rather, we emphasize that PMS implantation is commonly accompanied by PMS-specific perioperative practices and early postoperative decision points (e.g., early tissue–device interactions affecting outflow and the use and subsequent removal of intraluminal stenting in selected eyes). These considerations can influence when AC reformation alone may be sufficient versus when filtration-modulating interventions (e.g., re-stenting or compression sutures) should be considered, supporting the clinical need for a pragmatic, stepwise outpatient framework focused on PMS.

Although limited by the small sample size, the inclusion of only pseudophakic eyes, and the relatively short follow-up duration, this case series builds on previous studies by focusing specifically on hypotony associated with PMS implantation and by presenting a pragmatic, device-specific management framework.^1, 4)^ Unlike traditional filtering procedures, the PMS relies on a fixed outflow resistance determined by its dimensions. Consequently, the clinical course of hypotony and the response to OVD reinflation may differ in timing and durability depending on perioperative factors (including stenting practices and early tissue response), supporting the need for a pragmatic, stepwise framework. Because this was a retrospective case series, the timing of conservative treatment and OVD reinflation was not identical across cases, and this variability may have influenced short-term response and durability. Case 1 was included because the hypotony had early postoperative onset; however, definitive OVD reinflation at our institution was performed later in the course after delayed referral. Therefore, the applicability of this approach to similarly protracted cases requires further study. Finally, the impact of OVD injection on long-term IOP control and bleb function requires extended follow-up.

## 8 Conclusion

In conclusion, our three PMS cases suggest that small-volume intracameral high-viscosity OVD reinflation can serve as a simple, reproducible, and minimally invasive first-line option for Seidel-negative hypotony with shallow AC and CD, rapidly restoring chamber depth without compromising short-term IOP control. Implementing a stepwise management framework with prespecified escalation options for recurrent or severe cases may provide a practical approach for standardizing care as PMS use expands worldwide. This approach has the potential to streamline decision-making in busy clinical settings; however, larger prospective studies with longer follow-up are needed to confirm long-term corneal and macular safety and to refine patient selection and dosing parameters.

## CRediT authorship contribution statement

**Miho Takenaka:** Writing – original draft, Investigation, Data curation. **Yasuko Ikegami:** Data curation. **Miwako Yoshimoto:** Data curation. **Rei Sakata:** Writing – review & editing, Writing – original draft, Methodology, Investigation, Formal analysis, Conceptualization.

## Patient consent

The patients provided written informed consent for publication of the case details, clinical images, and the supplemental surgical video.

## Authorship

All authors attest that they meet the current ICMJE criteria for authorship and agree to be accountable for all aspects of the work.

## Claim of priority

After conducting a literature review on 2 December 2025 utilizing PubMed, Google Scholar, and Ichushi-Web with the key words “PreserFlo MicroShunt”, “hypotony”, “shallow anterior chamber”, “choroidal detachment”, and “ophthalmic viscosurgical device”, we did not find any prior reports describing a stepwise outpatient approach using small-volume intracameral OVD reinflation for early Seidel-negative hypotony after PreserFlo MicroShunt implantation.

## Consent

Written informed consent for publication of clinical images was obtained from the patient.

## Funding

No funding or grant support.

## Declaration of competing interest

The authors declare that they have no known competing financial interests or personal relationships that could have appeared to influence the work reported in this paper.

## Appendix A Supplementary data

Supplementary Video 1 Microscope view of intracameral ophthalmic viscosurgical device (OVD) reinflation for early Seidel-negative hypotony after PreserFlo MicroShunt (PMS) implantation. The video shows an outpatient procedure in a pseudophakic glaucoma eye that developed hypotony (intraocular pressure [IOP] 3 mmHg) with a shallow anterior chamber (AC) and choroidal detachment (CD) in the early postoperative period. After topical anesthesia and paracentesis creation, a fixed initial small volume (approximately 0.2 mL) of high-viscosity sodium hyaluronate is injected into the AC to deepen the chamber and restore pressurization. The final retained intraocular amount may vary depending on egress during chamber reformation. The video is playing at double speed.
